# A16 ESOPHAGEAL ORGANOID PROLIFERATION AND DIFFERENTIATION ARE ALTERED BY LOSS OF MSH2

**DOI:** 10.1093/jcag/gwab049.015

**Published:** 2022-02-21

**Authors:** M Rolland, A Gonneaud, D Jean, V Giroux

**Affiliations:** Department of Anatomy and Cell Biology, Faculty of Medicine and Health Sciences, Université de Sherbrooke, Sherbrooke, QC, Canada

## Abstract

**Background:**

The stratified epithelium of the esophagus includes *Krt15+* basal stem cells that display self-renewing and regenerative capacity, and multipotency. However, the mechanisms that specifically control their functions remain unknown. Interestingly, RNA sequencing and GSEA revealed an enrichment of a gene set associated with DNA repair in *Krt15+* cells vs *Krt15*- cells. We also observed that *Msh2* (DNA mismatch repair pathway) is the most significantly upregulated gene in *Krt15+* stem cells.

**Aims:**

To determine the effect of Msh2 loss on self-renewal and differentiation of esophageal organoids.

**Methods:**

Esophageal epithelial cells were isolated from a wild-type mouse. Using flow cytometry, esophageal *Krt15+* (GFP+) and *Krt15*- (GFP-) cells were sorted from *Krt15-CrePR1 (R26*^*mT/mG*^) mice. All cell populations were grown as organoids and *Msh2* was depleted using a CRISPR/Cas9 approach. Impact of *Msh2* loss on self-renewal and differentiation in esophageal epithelial organoids was evaluated through organoid formation assays, WST-1 proliferation assays and histological analysis.

**Results:**

At baseline, organoids depleted for *Msh2* formed more poorly differentiated and less well-differentiated organoids than controls. Lower expression of differentiation gene *Krt13* was also observed in *Msh2*-depleted organoids, confirming an altered differentiation pattern. Furthermore, these organoids showed a higher organoid formation rate and proliferation by WST-1 assay, suggesting that self-renewal capacity and viability are increased when *Msh2* is depleted. Interestingly, following radiation, organoids depleted for *Msh2* showed higher residual levels of p-H2AX (DNA damage marker), suggesting that their capacity to cope with DNA damages is altered. As mentioned above, we previously reported that *Msh2* is the most upregulated gene in *Krt15+* vs *Krt15*- cells. Therefore, to determine if Msh2 role is distinct in both populations, we depleted *Msh2* in *Krt15+* and *Krt15*- cells-derived organoids. Interestingly, our preliminary results suggest that *Msh2* deletion led to increased p-H2AX and decreased Krt13 levels in *Krt15+* organoids but not in *Krt15*- organoids.

**Conclusions:**

Our results show that Msh2 is potentially a key contributor of esophageal stemness in homeostatic and injured conditions.

**Funding Agencies:**

CIHRCanada Research Chair

